# Anti-inflammatory Effect of *Curcuma longa* and *Allium hookeri* Co-treatment via NF-κB and COX-2 Pathways

**DOI:** 10.1038/s41598-020-62749-7

**Published:** 2020-03-31

**Authors:** Soon-Young Lee, Seung-Sik Cho, YongChun Li, Chun-Sik Bae, Kyung Mok Park, Dae-Hun Park

**Affiliations:** 1 Department of Korean Medicine, Dongshin University, Naju, 58245 Korea; 2 Department of Pharmacy, College of Pharmacy, Mokpo National University, Muan Jeonnam, 58579 Korea; 3 School of Pharmaceutical Science, Zhengzhou University, Zhengzhou, Henan 450001 P.R. China; 4 College of Veterinary Medicine, Chonnam National University, Gwangju, 61186 Korea

**Keywords:** Drug discovery, Medical research

## Abstract

Although inflammation is a host defense mechanism, chronic inflammation mediates several diseases, including cancer, allergy, asthma, and autoimmune diseases, and reportedly, it is associated with a 60% mortality rate. There are several reports on the anti-inflammatory effects of *Curcuma longa* and *Allium hookeri*. However, although they can be used as culinary materials and have biological effects, they are not effective anti-inflammatory agents. In this study, we evaluated the synergic effect of *C. longa* and *A. hookeri* in order to confirm the possibility of a new anti-inflammatory agent. Based on cell viability and cytokine analyses, the appropriate ratio of *C. longa* and *A. hookeri* was confirmed using an air pouch animal model. Then, the anti-inflammatory effect of *C. longa* and *A. hookeri* co-treatment was evaluated by measuring the immune cell count and cytokines in the exudate and by comparing the morphological changes and cytokines in inflamed skin samples. Additionally, we evaluated the NF-κB/COX-2 pathway and iNOS levels. The active constituents detected in *C. longa* were demethoxycurcumin and bisdemethoxycurcumin, and that detected in *A. hookeri* was methylsulfonylmethane. An *in vitro* assessment determined the appropriate drug ratio as 3:7. In a carrageenan-induced inflammatory model, co-treatment effectively suppressed inflammatory cytokines, including IFN-γ, IL-1β, IL-6, IL-13, and IL-17, and recovered inflammation-related morphological changes in the skin. The anti-inflammatory effect of the co-treatment was mediated through the NF-κB/COX-2 pathway and iNOS inhibition. We concluded that co-treatment with *C. longa* and *A. hookeri* synergistically inhibited inflammation via the NF-κB/COX-2/iNOS pathway.

## Introduction

Inflammation is a biological, homeostatic defense mechanism against foreign bodies. However, chronic inflammation can cause additional damage in cases of cancer, allergy, asthma, autoimmune diseases, glomerulonephritis, hepatitis, inflammatory bowel disease, rheumatoid arthritis, and other disorders^1^. Moreover, chronic inflammation can result in death. Reportedly, chronic inflammatory diseases^2^ are associated with a 60% worldwide mortality. Steroidal hormones are the most potent anti-inflammatory drugs owing to their ability to block all inflammatory pathways; however, tolerance against such drugs is easily developed. Hence, non-steroidal anti-inflammatory drugs (NSAIDs) are broadly used. However, NSAIDs have severe adverse effects as they damage the upper gastrointestinal tract by inhibiting prostaglandin synthesis^3^.

Several cytokines influence the occurrence or inhibition of inflammation. Pro-inflammatory cytokines that promote inflammation include interleukin-1β (IL-1β), IL-6, IL-13, and tumor necrosis factor-alpha (TNF-α)^4^. Conversely, anti-inflammatory cytokines that can inhibit inflammation include IL-1Rα, IL-4, IL-10, IL-11, and TGF-β1^5^. Furthermore, the nuclear factor kappa B (NF-κB)/cyclooxygenase 2 (COX-2)/inducible nitric oxide synthase (iNOS) pathway is important in the pathogenesis of inflammation^6,7^.

Lately, there is an increasing number of trials attempting to develop safer and more effective anti-inflammatory drugs^8^. *Curcuma longa* (*C. longa*), a member of the Zingiberaceae family, has several biological effects such as anti-inflammation, hyperlipidemia inhibition, and gastroprotection^9–12^. Our group has also worked to develop safe anti-inflammatory drugs from natural products^13–15^. Previously, we reported the anti-inflammatory effect of *A. hookeri* but were unable to elucidate the exact mechanism of action involved in the effect^14^. In the present study, we investigated the mechanism of action of *A. hookeri* and *C. longa* co-treatment and evaluated their synergistic anti-inflammatory effect.

## Results

### Analysis of the active compounds in *C. longa* and *A. hookeri* extracts

**Figure 1:**
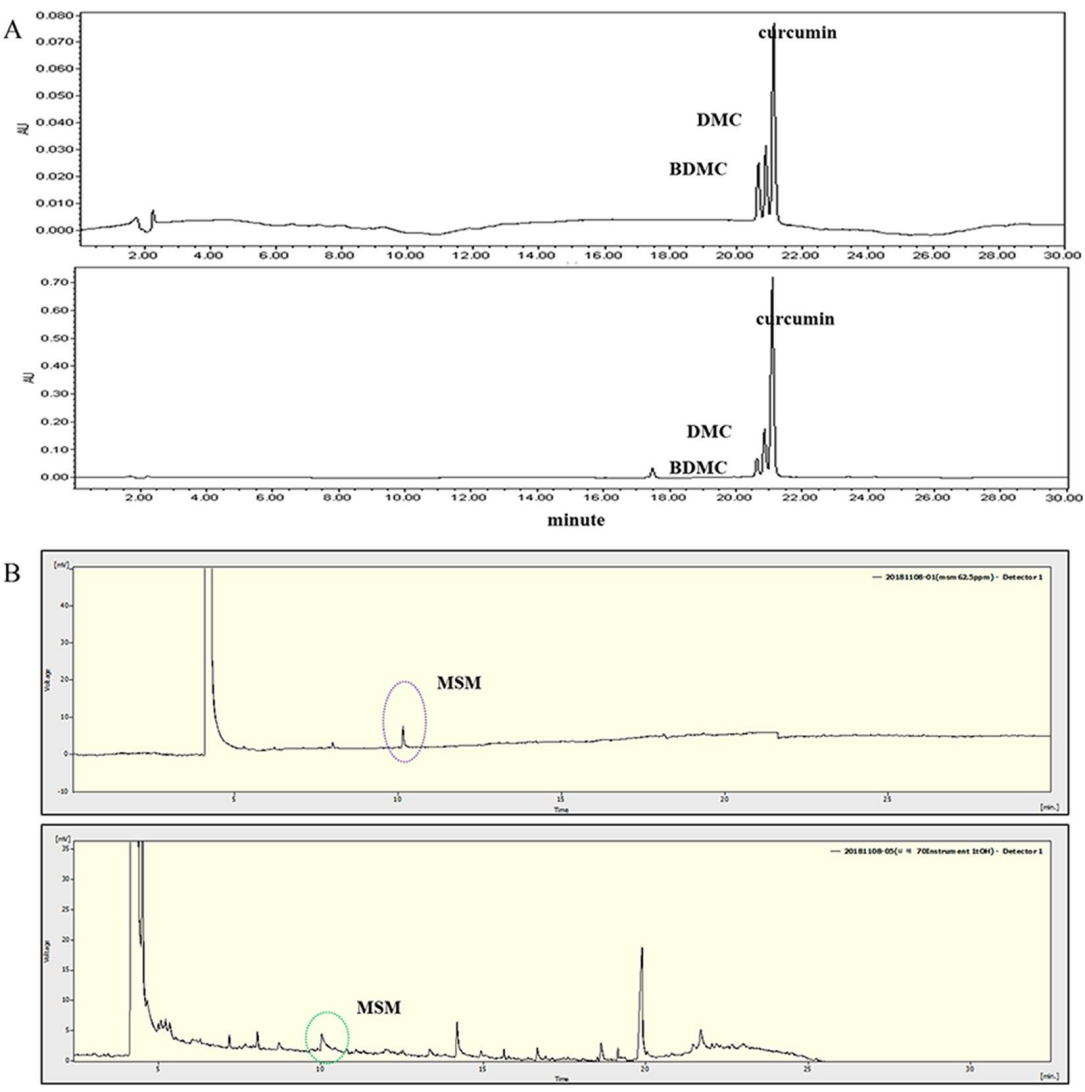
Identification of active compounds in *C. longa* extract and 70% ethanol extract of *A. hookeri* root. (**A**) At a retention time of 20 to 22 min, the active constituents in *C. longa* extract such as bisdemethoxycurcumin (BDMC), demethoxycurcumin (DMC), and curcumin, were detected via HPLC analysis. The upper graph is the standard at 450 nm and the lower graph is the sample extract at 450 nm. (**B**) At a retention time of 10 min, methylsulfonylmethane (MSM) in the 70% ethanolic extract of *A. hookeri* was confirmed via GC analysis. The upper graph is the standard and the lower graph is the sample extract. DMC, demethoxycurcumin; BDMC, Bisdemethoxycurcumin. MSM, methylsulfonylmethane.

The active constituents in both extracts were identified, and the representative chromatograms of the standard mixture and sample extracts are shown in Fig. 1. Curcumin and its two derivatives were the main components identified in the *C. longa* extract. The percentages of curcumin, demethoxycurcumin (DMC), and bisdemethoxycurcumin (BDMC) in the *C. longa* extract were 0.17, 0.11, and 0.06% (w/w), respectively. Methylsulfonylmethane (MSM) was identified in the A. hookeri extract, and its content (1%) was evaluated using gas chromatography. MSM is a well-known organic sulfur-containing compound found in small amounts in milk, grains, meat, eggs, and fish^16^.

### The ratio of *C. longa* vs. *A. hookeri* suppressing LPS-induced RAW 264.7 cell proliferation was found to be 3:7

**Figure 2:**
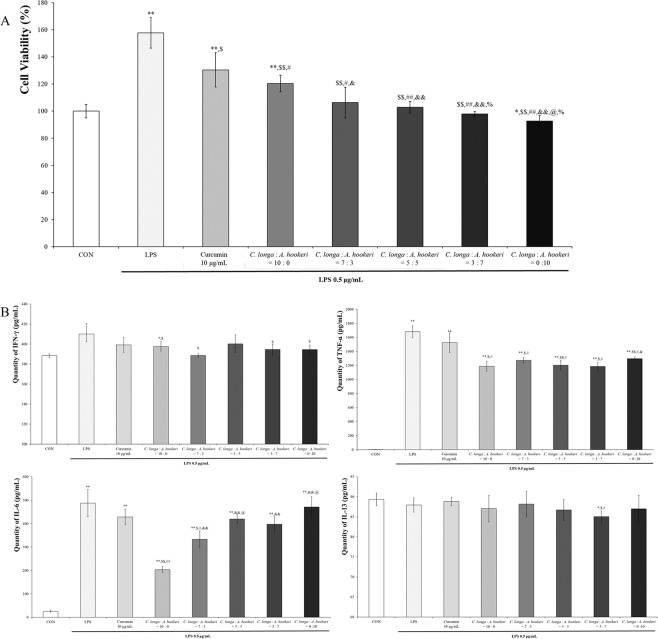
Cell proliferation suppression and pro-inflammatory cytokine regulation depending on the ratio of *C. longa* to *A. hookeri*. (**A**) Including curcumin alone treatment, the ratios of *C. longa* to *A. hookeri* were 10:0, 7:3, 5:5, 3:7, and 0:10, and controlled LPS-induced cell proliferation comparable to non-LPS-treated cells. The ratios (7:3, 5:5, 3.7, and 0:10) suppressed cell proliferation and the minimum ratio of cellular proliferation inhibition was 7:3. (**B**) In most groups, except the curcumin alone treatment group and the 5:5 ratio, IFN-γ levels, increased by LPS treatment, were suppressed by *C. longa* and *A. hookeri* co-treatment. Although the 7:3 ratio was most effective in suppressing IFN-γ expression, the ratios 7:3, 3:7, and *A. hookeri* alone downregulated IFN-γ levels. Unlike *A. hookeri, C. longa* dose-dependently inhibits IL-6 levels. The *C. longa* alone treatment most significantly inhibited IL-6 expression, but the 7:3, 5:5, or 3:7 ratios effectively suppressed IL-6 expression. IL-13 levels decreased only by the 3:7 *C. longa* to *A. hookeri*. In all groups, TNF-α levels were suppressed, with the lowest level by the 3:7 ratio. All values are presented as mean ± standard deviation. CON vs. ^*^p < 0.05; CON vs. ^**^p < 0.001 LPS vs. ^$^*p* < 0.05; LPS vs. ^$$^*p* <0.001; *C. longa*: *A. hookeri* = 10:0 vs. ^#^*p* < 0.05; *C. longa*: *A. hookeri* = 10:0 vs. ^##^*p* < 0.001; *C. longa*: *A. hookeri* = 7:3 vs. ^&^*p* < 0.05; *C*^.^ *longa*: *A. hookeri* = 5:5 vs. ^@^*p* < 0.05; *C. longa*: *A. hookeri* = 3:7 vs. ^%^*p* < 0.05.

In order to determine the effective ratio of *C. longa* and *A. hookeri* as well as curcumin alone (10 μg/mL) against LPS treatment-induced RAW 264.7 cell proliferation, serial concentrations of *C. longa* were used as shown in Fig. 2A. Depending on the increment of the relative concentration of *A. hookeri*, the cell viability was similar to that of the control group (100%). In order to evaluate the synergic effect of *C. longa* and *A. hookeri*, curcumin, the active constituent in *C. longa*, was analyzed. However, as the percentage of whole curcumins (includes curcumin metabolites) in *C. longa* was 0.34% (w/w) and 0.85 μg/mL whole curcumins existed in 250 μg/mL 30% EtOH-extracted *C. longa* (Supplementary File), and taking into consideration *C. longa* and *A. hookeri* combination treatment, the contribution effect of curcumin alone was confirmed (Fig. 2 and Supplementary File). At first, in order to investigate the applied dose of curcumin alone, cell viability was evaluated after treatment with curcumin alone (Supplementary File), and although whole curcumin existed at an amount of 0.85 μg/mL in *C. longa* alone, 10 μg/mL curcumin was used for the treatment, for confirmation of its anti-inflammatory effect. However, the effect differed significantly when the levels of pro-inflammatory cytokines were compared (Fig. 2B). The most effective ratios against each cytokine were variable: 7:3 against IFN-γ, 10:0 against IL-6, 3:7 against IL-13, and 3:7 against TNF-α. Furthermore, although the most suppressive ratios were 7:3 against IFN-γ and 10:0 against IL-6, 3:7 of *C. longa and A. hookeri* statistically suppressed IFN-γ and IL-6 levels.

### *C. longa and A. hookeri* co-treatment restored immune-related white blood cells in the exudate and completely recovered carrageenan-induced altered morphology in the skin via pro-inflammatory cytokine regulation

**Figure 3:**
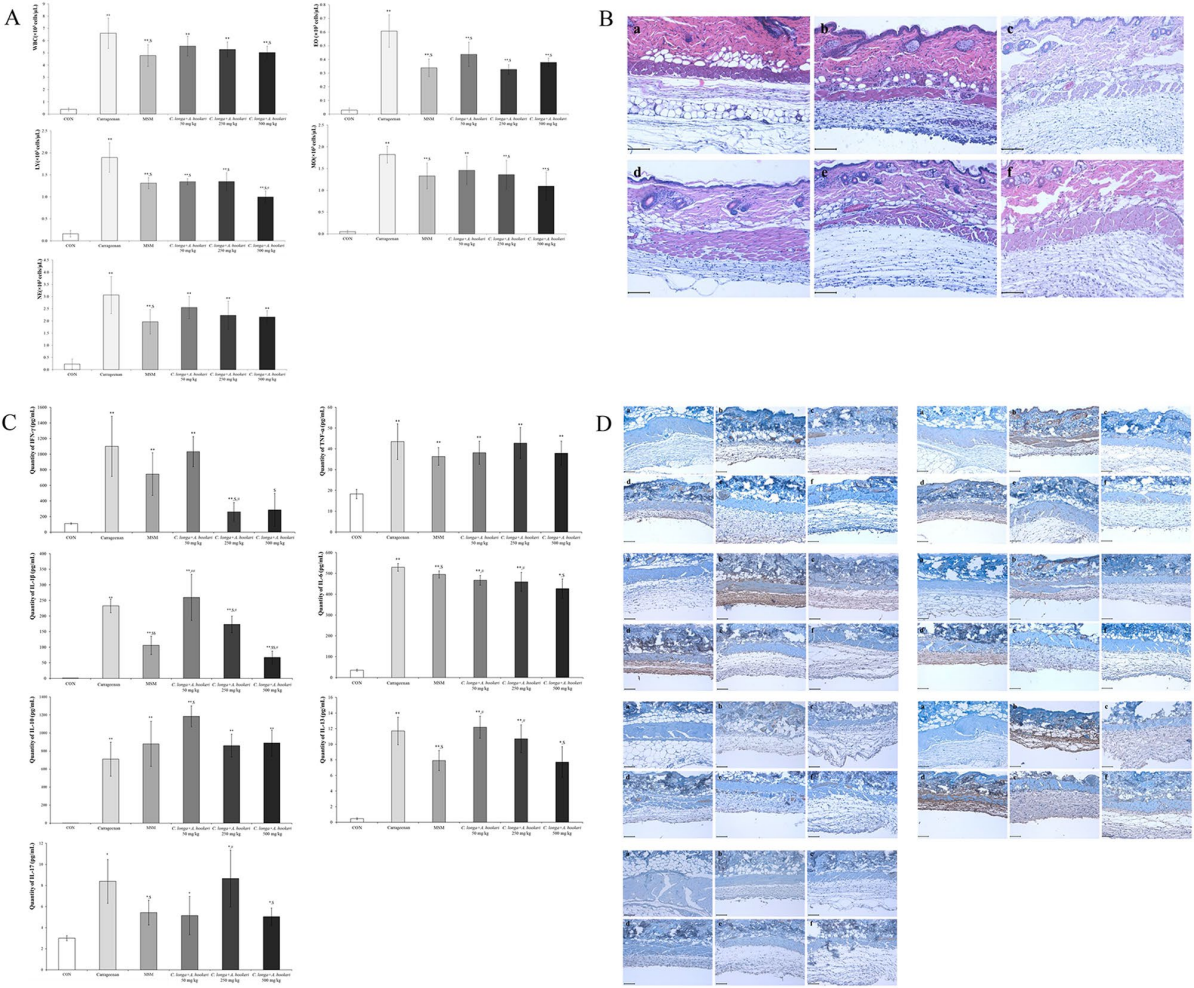
*C. longa* and *A. hookeri* co-treatment suppresses carrageenan-stimulated inflammation. (**A**) Inhibitory effect of co-treatment against blood cell proliferation in the exudate. Co-treatment with *C. longa* and *A. hookeri* dose-dependently inhibits white blood cells and monocyte proliferation, controls eosinophils levels similar to MSM treatment, completely decreases carrageenan treatment-upregulated lymphocyte levels, dose-dependently controls monocyte levels, and only demonstrates a tendency for neutrophil suppression with no statistical comparison. (**B**) Recovery effect of co-treatment on the air pouch membrane’s morphological changes. (**C**) Co-treatment suppresses the expression of inflammatory-related cytokines such as IFN-γ, IL-1β, IL-6, IL-13, and IL-17, but not TNF-α. Co-treatment with ≥250 mg/kg *C. longa* and *A. hookeri* exerts a stronger inhibitory effect on IFN-γ levels than MSM treatment. Co-treatment dose-dependently suppresses IL-1β, IL-6, and IL-13 levels. However, no significant decrease in TNF-α and IL-10 expression was observed with co-treatment. In the case of IL-17, there is a dose-dependent decrease pattern, which is hard to obtain with co-treatment. (**D**) Significant inhibitory effects against inflammatory-related cytokines in the dermis such as IFN-γ, TNF-α, IL-1β, IL-6, IL-13, and IL-17. Co-treatment dose-dependently suppresses the expression of cytokines such as IFN-γ, IL-1β, IL-10, and IL-17, and 500 mg/kg treatment completely inhibits the expression of IFN-γ, IL-10, and IL-17. In a dose-dependent manner, the levels of IL-1β, IL-6, and IL-13 are effectively downregulated. Co-treatment effectively inhibits the expression of TNF-α, with complete suppression observed with 250 mg/kg and 500 mg/kg co-treatment. a, control; b, 1% carrageenan treatment; c, 25 mg/kg MSM treatment; d, 50 mg/kg *C. longa* and *A. hookeri* co-treatment; e, 250 mg/kg *C. longa* and *A. hookeri* co-treatment; f, 500 mg/kg *C. longa* and *A. hookeri* co-treatment. Magnification, 200×. Scare bar, 100 μm. All values are presented as mean ± standard deviation. CON vs. ^**^p < 0.001; Carrageenan vs. ^$^*p* < 0.05; MSM vs. ^#^*p* < 0.05.

With the exception of neutrophils, *C. longa and A. hookeri* co-treatment downregulated white blood cells, eosinophils, lymphocytes, and monocytes in a dose-dependent manner (Fig. 3A). Although the population of neutrophils was not regulated in a dose-dependent manner, a downregulation was observed. To measure the anti-inflammatory effect of the co-treatment, skin morphology was analyzed (Fig. 3B). Carrageenan changed skin morphology by inducing inflammation, which resulted in a thin membrane, muscle condensation, downregulation of adipocytes, and deep wrinkles, in comparison to the normal group. However, MSM and co-treatment ameliorated the altered skin membrane morphology, with *C. longa* and *A. hookeri* co-treatment dose-dependently restoring the altered skin membrane. Carrageenan stimulated inflammation-related cytokines such as IFN-γ, TNF-α, IL-1β, IL-6, IL-10, IL-13, and IL-17. IFN-γ is a pro-inflammatory cytokine and its expression increases during inflammatory conditions (Fig. 3C,D). Inflammation-related cytokines in the exudate, increased by carrageenan treatment, were regulated by co-treatment (Fig. 3C). The levels of IFN-γ, IL-1β, IL-6, IL-13, and IL-17 were downregulated by co-treatment; however, those of TNF-α and IL-10 were not suppressed. Notably, the level of IFN-γ decreased to the control level following co-treatment. Similarly, carrageenan stimulated IFN-γ and IL-1β expression in the exudate. However, with high dose co-treatment (500 mg/kg), IFN-γ and IL-1β levels were reduced to levels similar to those in the control group. Co-treatment dose-dependently suppressed the levels of IL-6, IL-13, and IL-17, which were upregulated by carrageenan. IL-10 was not suppressed by the treatment. In the case of TNF-α, the results obtained for the exudate and skin differed. Although TNF-α level was not suppressed by co-treatment in the exudate, its expression was completely inhibited in the skin of the animals in the 500 mg/kg co-treatment group.

### *C. longa and A. hookeri* co-treatment effectively controlled both carrageenan-induced inflammations by regulating the NF-κB–COX-2 pathway and iNOS level

**Figure 4:**
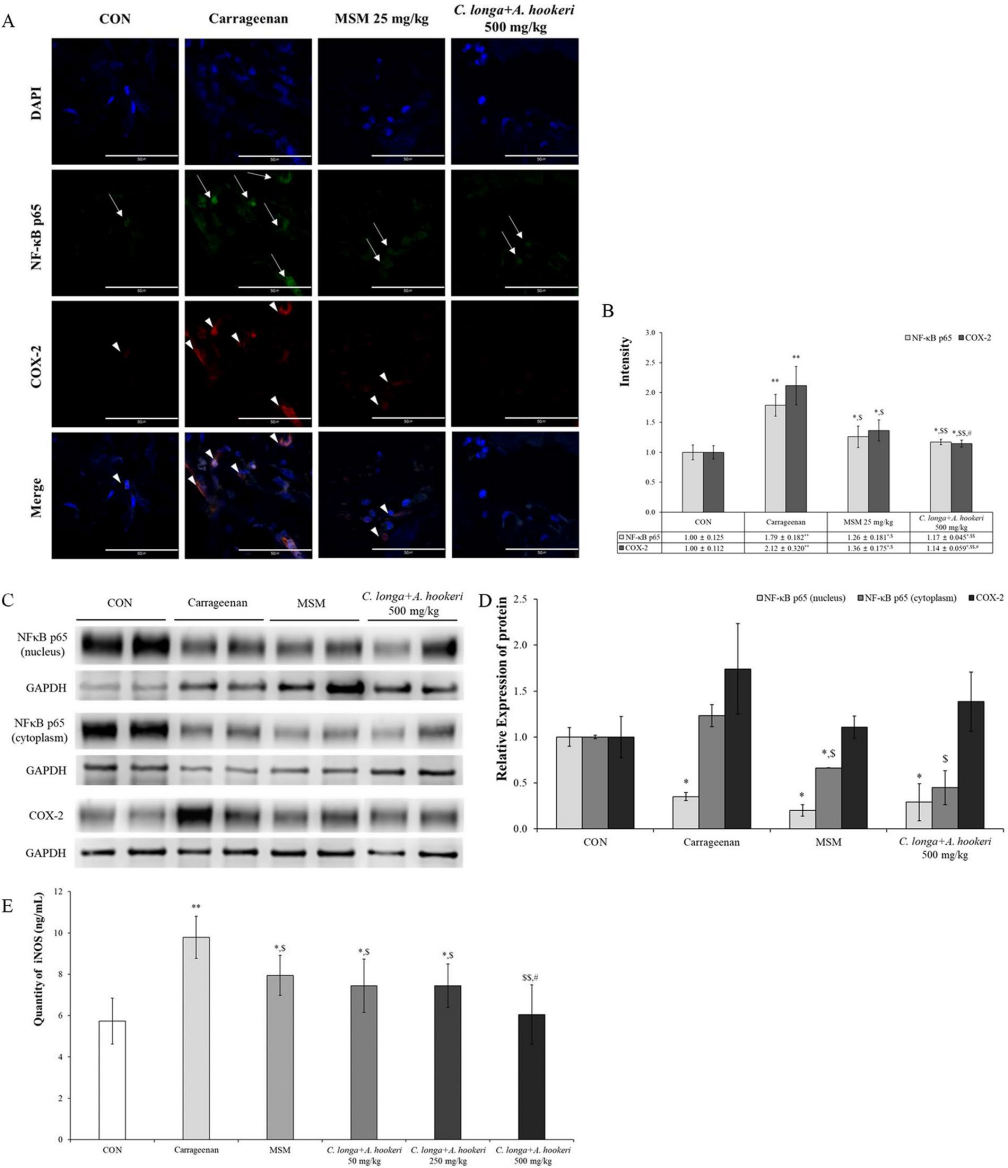
*C. longa* and *A. hookeri* co-treatment inhibits NF-κB and COX-2 expression. (**A**) NF-κB is downregulated in the nucleus and COX-2 in the cytoplasm. NF-κB and COX-2 are not expressed in the control group and only the nucleus is stained with DAPI (blue spots). Carrageenan stimulates the expression of NF-κB in the nucleus and that of COX-2 in the cytoplasm. MSM suppresses the expression of NF-κB and COX-2 induced by carrageenan, but the expression of NF-κB (arrow) and COX-2 (arrowhead) observed is minimal. *C. longa* and *A. hookeri* co-treatment significantly inhibits the expression of NF-κB and COX-2. (**B**) The graph and scores present the immunofluorescent results using the image analyzing program in the K1-Fluo confocal microscope. MSM and *C. longa* and *A. hookeri* co-treatment statistically significantly control NF-κB and COX-2 expression effectively. (**C**) Compared to the density of NF-κB in the nucleus and cytoplasm, the pattern is similar to that observed in the immunofluorescence assay. *C. longa* and *A. hookeri* co-treatment blocks the translocation of NF-κB into the nucleus from the cytoplasm and inhibits the expression of COX-2 in the cytoplasm. (**D**) Depending on the image analysis of the western blotting of NF-κB (in nucleus and in cytoplasm) and COX-2 (in the skin tissue), the density graph based on each band per β-actin was presented using the Image J program. (**E**) MSM and *C. longa* and *A. hookeri* co-treatment significantly inhibited carrageenan-induced iNOS in the serum. The downregulation effect of 500 mg/kg *C. longa* and *A. hookeri* co-treatment is similar to that of the control. All values are presented as mean ± standard deviation. Arrow, NF-κB; arrowhead, COX-2. Magnification, 1000×. Scare bar, 50 μm. CON vs. ^*^p < 0.05; CON vs. ^**^p < 0.001 Carrageenan vs. ^$^*p* < 0.05; Carrageenan vs. ^$$^*p* < 0.001; MSM vs. ^#^*p* < 0.05. **Table in** Fig. 4**. The ratio of NF-κB p65 vs. nucleus or COX-2 vs. nucleus**. All values are presented as mean ± standard deviation. CON vs. ^*^p < 0.05; CON vs. ^**^p < 0.001; Carrageenan vs. ^$^*p* < 0.05; Carrageenan vs. ^$$^*p* < 0.001; MSM vs. ^#^*p* < 0.05.

Changes in NF-κB and COX-2 were assessed using the immunofluorescent method (Fig. 4). The results were analyzed by the image analyzing program in the K1-Fluo confocal microscope (Fig. 4A). Carrageenan increased NF-κB levels in the nucleus (1.79 ± 0.182) compared to those in the control group (1.00 ± 0.125); however, MSM suppressed the carrageen-induced increase in NF-κB (1.26 ± 0.181) (Fig. 4B). The decrease in the level of NF-κB in the *C. longa* and *A. hookeri* co-treatment group (1.17 ± 0.045) was comparable with that observed in the MSM treatment group. In the carrageenan treatment group (2.12 ± 0.320), the cytoplasmic level of COX-2 significantly increased compared to that in the control group (1.00 ± 0.112). In the co-treatment group, the expression of COX-2 was minimal (1.14 ± 0.059). The level of NF-κB in the nucleus or cytoplasm was evaluated using western blotting (Fig. 4C,D), and the level of NF-κB in the nucleus significantly increased compared to that in the carrageenan treatment group both inthe MSM treatment group and in 500 mg/kg *C. longa* and *A. hookeri* co-treatment group, nuclear and cytoplasmic NF-κB levels decreased. In order to measure the anti-inflammatory effect and mechanism of co-treatment, serum iNOS levels were measured (Fig. 4E). Co-treatment dose-dependently suppressed the carrageenan-induced serum iNOS level. Particularly, in the 500 mg/kg co-treatment group, iNOS levels were lower than those observed in the MSM treatment group and similar to those in the control group.

## Discussion

Inflammation is a homeostatic response in organisms against various conditions. However, when this response is excessive, it could result in conditions such as atherosclerosis, rheumatoid arthritis, and asthma^17–19^. Hence, there are several ongoing trials aimed at decreasing the occurrence of excessive inflammation. Several cytokines are related to the inflammatory process. Pro-inflammatory cytokines include IL-1β, IL-6, IL-13, and TNF-α^4^. IFN-γ is an important cytokine that stimulates cell-mediated immune reactions^20^ and regulates inflammatory diseases as a pro-inflammatory cytokine. However, reports contrary to the induction of inflammation have been reported, and the yin and yang theory of IFN-γ has been published^21^. For example, during IFN-γ-mediated inflammation control, the downregulatory effect of IFN-γ was induced by an increase in programmed cell death 1 (PD-1) and PD-1 ligand 1 (PD-L1) in the rheumatoid synovium;^22^ however, an upregulatory effect was induced by the antagonizing IL-17 function^23,24^. Additionally, TNF-α is released by macrophage and neutrophil activation induced by IFN-γ^25^. Thus, IFN-γ demonstrates a “double-edged sword” effect; it induces inflammatory diseases such as rheumatoid arthritis and inflammatory bowel disease^26,27^ related to TNF receptor 1, and it is involved in homeostasis, for example, survival and regeneration of cells or tissues through TNF receptor 2^28^. IL-6 activation has a pivotal role in inflammation and inflammation-induced cell damage. Hence, IL-6 modulation has been used as a target for anti-inflammatory drug development, including drugs against rheumatoid arthritis and juvenile idiopathic arthritis^29^. IL-13 is a key cytokine in allergic and inflammatory diseases that induces an inflammatory cascade^30^. IL-13 is a drug target in several inflammatory diseases, including asthma, atopic dermatitis, and inflammatory bowel disease^31^.

There are several known causes of inflammation, and the NF-κB/COX-2/iNOS pathway is a known important mechanism^6,7^. Notably, NF-κB controls various stages of inflammation and immune modulation via the regulation of several molecules, including cytokines (e.g., IL-1β, TNF-α), iNOS, and chemokines^32,33^.

*C. longa* has been used as a culinary material worldwide and curcumin is one of its well-known active components. It is commonly used as a spice, food additive, or dietary pigment. Curcumin has been known to have several pharmacological effects, including anti-inflammatory, antioxidant, and anti-cancer activities^34–36^. The molecular mechanisms of its effects are diverse, involving various signaling pathways (such as NF-κB and STAT3 signaling)^37^. Thus, curcumin and other *C. longa* components are promising inflammation-targeting phytochemicals. Sandur *et al*.^38^ reported that curcumin, demethoxycurcumin, and bisdemethoxycurcumin are the active substances in *C. longa* that suppressed TNF-induced NF-κB activation. Their effects were found to be due to the methoxy groups on the phenyl ring.

*A. hookeri* has been cultivated for consumption in East Asia. MSM is an active constituent of *A. hookeri*, and known to have anti-inflammatory, anti-arthritic, anti-allergic, and anti-asthmatic effects^16,39,40^. Kim *et al*. reported that MSM inhibits nitric oxide prostaglandin 2 production via suppressing iNOS and COX-2 expression. Moreover, MSM strongly inhibits IL-6 and TNF-α production through the transcription factor NF*-κ* B^16^.

In the present study, we identified curcumin and its two derivatives in *C. longa* extract. Curcumin is a well-known bioactive ingredient, reported to have a synergistic effect with various bioactive components. Morgana *et al*. reported that curcumin and piperine increased TGF-β levels, significantly improved collagen repair, and decreased cellularity and activation of NF-ĸB in periodontal tissues^41^. Nishtha *et al*. found that curcumin and quercetin synergistically inhibit cancer cell proliferation and modulate Wnt signaling pathways in cancer cells^42^. Chen *et al*. conducted a study similar to ours. They elucidated the synergistic anti-inflammatory effects of a curcumin, tetramethylpyrazine, and resveratrol mixture^43^. Based on previous reports, curcumin and other *C. longa* extract substances exert synergistic effects in combination with *A. hookeri* extract. For future studies, the interaction between the main bioactive substances of *C. longa* and the single substance present in *A. hookeri*, should be investigated.

We previously reported that the anti-inflammatory efficacy of *A. hookeri* is via the regulation of pro-inflammatory cytokines such as IL-1β, IL-6, IL-13, and TNF-α^14^. In our previous study, we demonstrated that 300 mg/kg *A. hookeri* treatment suppressed carrageenan-induced inflammation, with notable downregulation of skin pro-inflammatory cytokine levels. However, in this study, we observed a synergistic effect of *C. longa* and *A. hookeri* co-treatment. Although the 50 mg/kg *C. longa* and *A. hookeri* co-treatment (3:7) consisted of only 35 mg/kg *A. hookeri* extract, the levels of several inflammation-related cytokines, including IFN-γ, IL-1β, IL-6, IL-13, and IL-17, were suppressed (Fig. 3D). Specifically, 250 mg/kg co-treatment (175 mg/kg *A. hookeri*) significantly controlled TNF-α expression, and 500 mg/kg co-treatment (350 mg/kg *A. hookeri*) effectively suppressed IFN-γ and TNF-α levels. In the case of the exudate, 250 and 500 mg/kg co-treatment modulated IFN-γ levels to those observed in the control group, 500 mg/kg co-treatment effectively controlled IL-1β and IL-6 levels compared to those in the positive control (MSM), and IL-13 and IL-17 expression in the 500  mg/kg co-treatment were comparable to those observed with MSM treatment (Fig. 3C).

Based on the above results, we conclude that *C. longa* and *A. hookeri* co-treatment synergistically inhibit inflammation by regulating the NF-κB/COX-2/iNOS pathway.

## Materials and Methods

### Plant material preparation

Dried *C. longa* roots were obtained from the Chonnam Medical Herb-Agricultural Cooperation (Hwasun, Chonnam, South Korea). The extraction was performed 10 times with 30% ethanol at 80 °C for 1 h, and the extract was concentrated at 40 mmHg at 50 °C. The extract was pre-freezed in a deep freezer for 2 days as 2-L aliquots. Subsequently, after the extraction process, the extract was freeze-dried at 0.06 mbar at −70 °C for 48 h and stored at −50 °C. *A. hookeri* was supplied by the College of Pharmacy, Mokpo National University. The voucher specimen MNUCSS-SC-01 was recorded. Briefly, the root was separated for the study. Air-dried, powdered *A. hookeri* roots (1000 g) were extracted twice with 70% ethanol (4 L) at room temperature for 3 days. After filtration, ethanol evaporated, and the sample was freeze-dried and stored at 50 °C^14^.

The extraction was performed 10 times with either 70% ethanol (*A. hookeri*) or 30% ethanol (*C. longa*) at 80 °C for 1 h. The extract was concentrated at 40 mmHg at 50 °C. Pre-freezing of the high concentrated extract was performed in a deep freezer for 2 days by the 2-L aliquot respectively. Subsequently, after the extraction process, freeze-drying was carried out at 0.06 mbar at −70 °C for 48 h. The freeze-dried extract was stored at −20 °C with grinding and packing.

### Instrumentation and chromatographic conditions for *C. longa and A. hookeri*

All analysis for *C. longa* was performed using an Alliance 2695 HPLC system (Waters, Milford, MA, USA) equipped with a photodiode array detector^44^. An Agilent Zorbax extended C18 analytical column (5 µm, 150 mm × 5 mm) was used with a mobile phase consisting of a mixture of solvent A (acetonitrile) and B (water containing 0.2% phosphoric acid, pH 3.5). A gradient elution (20% A in B solvent ~ 100% A solvent) at a flow rate of 0.8 mL/min (Table 1) was employed. The detection wavelength was 450 nm. The solvent was filtered through a 0.22-µm filter and degassed. The sample injection volume was 10 µL. Agilent 7890 gas chromatography (GC) and Agilent 5975 quadrupole mass spectrometry (MS) system (Agilent Technologies, Palo Alto, CA, USA) were used to analyze molecular mass fragments of *A. hookeri* root^14^. The mass fragments were ionized in an Agilent DB-1 capillary column (30 m l. × 0.32 mm i.d., 0.25-µm film thickness) under electron ionization (EI) conditions. GC oven was thermally programmed as described in Table 2. All the scanned mass spectra were compared with the data system library (NIST 2017).

**Table 1:** Analytical HPLC conditions for *Curcuma longa* extract.

Parameters	Conditions		
Column	Zorbax extended-C18 (C18, 4.6 mm × 150 mm, 5 µm)		
Flow rate	0.8 mL/min		
Injection volume	10 μL		
UV detection	450 nm		
Run time	30 min		
Gradient	**Time (min)**	**% A**^1^	**% B**^2^
	0	20	80
	11	20	80
	25	100	0
	27	20	80
	30	20	80

^1^ Acetonitrile;^2^ 0.2% phosphoric acid (pH 3.5).

**Table 2:** Operation parameters for *Allium hookeri* extract.

Items	Gas chromatography
Column	DB-1 capillary column (0.32 mm I.D X 30 m, 0.25 μm, dimethylpolysiloxane, Agilent Tech, CA, USA)
Carrier gas	Nitrogen
Flow gas	1.0 mL/min
Injector Temp.	250 °C
Detector temp.	300 °C
Oven temp.	60 °C (4 min) → (8 °C/min) → 120 °C (3 min)
Split ratio	25:1
Injection volume	1 μL

### Evaluation of an appropriate ratio of *C. longa and A. hookeri*

To confirm the appropriate ratio of *C. longa* and *A. hookeri*, we conducted two measurements. We assessed their suppression effect against lipopolysaccharide (LPS)-induced proliferation of RAW 264.7 cells (Korean Cell Line Bank, Seoul, Korea), a murine macrophage cell line, and measured the pro-inflammatory cytokine levels at different ratios.

The suppression effect mediated by the *C. longa* and *A. hookeri* mixture was analyzed using 3-(4,5-dimethylthiazol-2-yl)-2,5-diphenyltetrazolium bromide (MTT; VWR Life Science, OH, USA) assay. On the first day, 1 × 10^4^ RAW 264.7 cells/well were seeded in 96-well plates, followed by treatment with LPS (Sigma-Aldrich, MO, USA) on the second day. On the third day, treatment-mediated cell proliferation was measured using the MTT assay.

To analyze the pro-inflammatory cytokine levels after each treatment, enzyme-linked immunoassays (ELISA) were conducted. To analyze the levels of IFN-γ, TNF-α, and IL-6 OptEIA, mouse ELISA kits were purchased from BD Biosciences. IL-1β, IL-10, IL-13, and IL-17 levels were assessed using mouse ELISA kits purchased from Thermo Fisher Scientific (MA, USA). All assays were conducted in accordance with the manufacturer’s guidelines.

### Carrageenan-induced air pouch model

The animal study was conducted twice using the same method. In each study, 36 male ICR mice (20–25 g) were purchased from Samtako Korea (Osan, Korea) and acclimatized for 8 days. To induce a subcutaneous air pouch from the ninth day, 3 mL of air was injected three times into the intra-scapular area for 6 days, and all mice were classified into two categories; the control mice (CON) were not treated with carrageenan, while the others received treatment. The carrageenan-treated category consisted of five groups including the normal saline oral administration group (Carrageenan), 25 mg/kg methylsulfonylmethane (MSM)-treated group (used as an anti-inflammatory drug), 50 mg/kg *C. longa* and *A. hookeri*-treated group, 250 mg/kg *C. longa* and *A. hookeri*-treated group, and 500 mg/kg *C. longa* and *A. hookeri*-treated group. In the animal study, the appropriate anti-inflammatory ratio of *C. longa* vs*. A. hookeri* was confirmed as 3:7. Two hours after the above treatments, 1 mL carrageenan solution (2%) was injected into the air pouch. After 24 h of the carrageenan injection, all mice were sacrificed using Zoletil (tiletamine HCl and zolazepam HCl; Virbac, Carros, France) via intraperitoneal injection. To collect the exudate, the pouches were flushed with 2 mL of phosphate-buffered saline. The number of total and differential cells in the pouch exudate were counted using the Hemavet Multispecies Hematology System (Drew Scientific Inc., Waterbury, CT, USA).

### Histopathological analysis

Histopathological measurement was conducted according to our previous study^14^. After exudate and blood collection, the skin tissues were retrieved, fixed in 10% (v/v) formaldehyde solution, dehydrated in a graded ethanol series (99.9%, 90%, 80%, and 70%), and embedded in paraffin. Paraffin-embedded skin tissues were then sectioned (5 µm) and stained with hematoxylin and eosin.

### Immunohistochemical analysis

Immunohistochemical analysis was performed according to our previous study^14^. Deparaffinized tissue sections were treated with 3% hydrogen peroxide in methanol for 10 min, to remove endogenous peroxidase. Antigen retrieval was performed with the sodium citrate buffer (0.1 M), using the boiling method. The slides were incubated with normal serum to prevent nonspecific binding and then incubated overnight at 4 °C with the following primary antibodies (diluted 1:100 or 1:200): IFN-γ (Santa Cruz, sc-74104), TNF-α (MY BioSource, CA, USA, MBS175453), IL-1β (Santa Cruz, sc-1251), IL-6 (Santa Cruz, sc-7920), IL-10 (Santa Cruz, sc-73309), IL-13 (Santa Cruz, sc-1776), and IL-17 (Abcam, MA, USA, ab79056). The slides were incubated for 2 h with biotinylated secondary antibody (1:500; DAKO, Carpinteria, CA, USA) and horseradish-peroxidase conjugated streptavidin. Signals were detected using the 3,3-diaminobenzidine tetrahydrochloride substrate chromogen solution, and cells were counterstained with Mayer’s hematoxylin.

### Immunofluorescence analysis

NF-κB and COX-2 expression levels were measured using the immunofluorescent method. NF-κB (ThermoFisher Scientific, PA5-16545, Waltham, MA, USA), COX-2 (Invitrogen, PA1-9032, Carlsbad, CA, USA), FITC-conjugated IgG (Jackson Immunoresearch, 315-095-003, West Grove, PA, USA), Alexa Fluor 555-conjugated IgG (ThermoFisher Scientific, A-21127), and DAPI (ThermoFisher Scientific, 62249) were used. A K1-Fluo Confocal Microscope (Nanoscope System, Daejeon, Korea) was used for image acquisition and for analyzing the fluorescent intensity.

### Western blotting

Each skin sample was prepared with 500 μL lysis buffer, centrifuged at 9,000 x g for 20 min, and the supernatant was collected. For NF-κB measurement, the nuclear or cytoplasmic proteins were separated by NE-PER Nuclear and Cytoplasmic Extraction Reagents (ThermoFisher Scientific, 78833), but COX-2 level was evaluated using the whole protein. For each group, 20 μg protein was diluted in SDS sample buffer, boiled, and electrophoresed on 10% acrylamide SDS-PAGE gels. Samples were transferred to nitrocellulose membranes and blocked with 5% skim milk overnight such that each protein in the sample could bind with primary antibodies in the 5% skim milk. After washing three times with Tris-buffered saline containing 0.1% Tween-20, the membranes were treated with peroxidase-conjugated secondary antibodies and visualized using ECL reagents. NF-κB (ThermoFisher Scientific, PA5-16545), COX-2 (Abcam, ab15191), GAPDH (Thermo Fisher Scientific, MA5-15738), and Peroxidase-conjugated Affinipure rabbit anti-mouse IgG (Jackson Immunoresearch, 315-035-003) were used.

### iNOS analysis

The animals were anesthetized and the exudate in their air pouches were collected. Blood samples were obtained from the heart and allowed to coagulate for 2 h at room temperature. Next, the samples were centrifuged at 1000×g for 15 min, and the serum iNOS levels were measured using a mouse iNOS ELISA kit (Mybiosource, MBS723353) according to the manufacturer’s guidelines.

### Ethics statement

All animals were maintained according to the guidelines of the Institutional Animal Care and Use Committee (IACUC) at Chonnam National University. This study was approved by Chonnam National University IACUC (Approval No.: CNU IACUC-YB-2019-47).

### Statistics

Results are expressed as mean ± standard deviation (SD). Group differences were evaluated using one-way analysis of variance, followed by Dunnett’s multiple comparison test. *p* < 0.05 or *p* < 0.001 was considered statistically significant.

## Supplementary information

Supplementary Information.

Supplementary Information2.

## References

[1] Philip H (2012). The inflammation theory of disease. EMBO Reports.

[2] Pahwa, R., Jialal, I., Chronic inflammation. StatPearls [Internet], https://www.ncbi.nlm.nih.gov/books/NBK493173 (June 4, 2019). 29630225

[3] Sostres C, Gargallo CJ, Arroyo MT, Lanas A (2010). Adverse effects of non-steroidal anti-inflammatory drugs (NSAIDS, aspirin and coxibs) on upper agstointestinal tract. Best Pract. Res. Clin. Gastroenterol..

[4] Zhang J-M, An J (2007). Cytokines, Inflammtion and pain. Int. Anestesiol. Clin..

[5] Musolino C (2017). Inflammatory and anti-inflammatory equilibrium, proliferative and antiproliferative balance: the role of cytokines in multiple myeloma. Mediators Inflamm..

[6] Surh Y-J (2001). Molecular mechanisms underlying chemopreventive activities of anti-inflammatory phytochemicals: down-regulation of COX-2 and iNOS through suppression of NF-κB activation. Mut. Res..

[7] Bulugonda RK (2017). Magniferin from Pueraria tuberosa reduces inflammation via inactivation of NLRP2 inflammasome. Sci Rep-UK.

[8] Devi VK, Jain N, Valli KS (2010). Imprtance of novel drug delivery systems in herbal medicines. Pharmacogn. Rev..

[9] Shehaz A, Rehman G, Lee YS (2013). Curcumin in inflammatory diseases. Biofactors.

[10] Henrotin Y (2010). Biological actions of curcumin on articular chondrocytes. Osteoarthritis Cartilage.

[11] Maithilikarpagaselvi N, Sridhar MG, Swaminathan RP, Sripradha R (2016). Curcumin inhibits hyperlipidemia and hepatic fat accumulation in high-fructose-fed male wistar rat. Pharm. Biol..

[12] Yadav SK (2013). Tumeric (curcumin) remedies gastroprotective action. Pharmacogn. Rev..

[13] Oh HN (2019). Tyrosinase inhibition antioxidant effect and cytotoxicity studies of the extracts of *Cudrania tricuspidata* fruit standardized in chlorogenic acid. Molecules..

[14] Kim JE (2017). Anti-inflammatory effect of *Allium hookeri* on carrageenan-induced air pouch mouse model. PloS One.

[15] Park DE (2017). Antimicrobial and anti-inflammatory effects of ethanol extract of *Corylopsis coreana* Uyeki Flos. Pharmacogn. Mag..

[16] Kim YH (2009). The anti-inflammatory effects of methylsulfonylmethane on lipopolysaccharide-induced inflammatory responses in murine macrophages. Biol. Pharm. Bull..

[17] Shoenfeld Y, Sherer Y, Harats D (2001). Artherosclerosis as an infectious, inflammatory and autoimmmune disease. Trends Immunol..

[18] Kourilovitch M, Galarza-Maldonado C, Ortiz-Prado E (2014). Diagnosis and classification of rheumatoid arthritis. J. Autoimmun..

[19] Durham AL, Caramori G, Chung KF, Adock IM (2016). Targeted anti-inflammatory therapeutics in ashtma and chronic obstructive lung disease. Transl. Res..

[20] Hoffmann G, Wirleitner B, Fuchs D (2003). Potential role of immune system activation-associated production of enopterin derivatives in human. Inflamm. Res..

[21] Zhang J (2007). Yin and yang interplay of IFN-γ in inflammation and autoimmune disease. J. Clin. Invest..

[22] Wan B (2006). Aberrant regulation of synovial T cell activation by soluble costimulatory molecules in rheumatoid arthritis. J. Immunol..

[23] Harrington LE (2005). Interleukin 17-producing CD4+ effector T cells develop via a lineasge distinct from the T helper type 1 and 2 lineages. Nat. Immunol..

[24] Sawitzki B (2005). IFN-gamma production by alloantigen-reactive regulatory T cells is important for their regulatory function *in vivo*. J. Exp. Med..

[25] Philip R, Epstein LB (1986). Tumor necrosis factor as immunomodulator and mediator monocyte cytotoxicity induced by itself, gamma-interferon and interleukin-1. Nature.

[26] Monaco C, Nanchahal J, Taylor P, Feldmann M (2015). Anti-TNF therapy: past, present and future. Int. Immunol..

[27] Lee RA, Eisen DB (2015). Treatment of hidradenitis suppurativa with biologic medications. J. Am. Acad. Dermatol..

[28] Probert L (2015). TNF and its receptors in the CNS: the essential, the desirable and the deleterious effects. Neurosicience.

[29] Tanaka T, Narazaki M, Kishimoto T (2014). IL-6 in inflammation, immunity, and disease. Cold Spring Harb. Perspect. Biol..

[30] Hoving JC (2018). Targeting IL-13 as a host-directed therapy against ulcerative colitis. Front. Cell. Infect. Microbiol..

[31] May RD, Fung M (2015). Strategies targeting the IL-4/IL-13 axes in disease. Cytokine.

[32] Lee GS, Choi KC, Han HJ, Jeung EB (2007). The classical and a non-classical pathways associated with NF-κB are involved in estrogen-mediated regulation of calbindin-D9k gene in rat pituitary cells. Mol. Cell. Endocrinol..

[33] Wei X (2015). Hydrogen sulfide inhalation improves neurological outcome via NF-κB-mediated inflammatory pathway in a rat model of cardiac arrest and resuscitation. Cell. Physiol. Biochem..

[34] Chandrasekaran CV (2013). Immune-stimulatory and anti-inflammatory activities of *Curcuma longa* extract and its polysaccharide fraction. Pharmacognosy Res..

[35] Uchio R, Murosaki S, Ichikawa H (2018). Hot water extract of turmeric (Curcuma longa) prevents non-alcoholic steatohepatitis in mice by inhibiting hepatic oxidative stress and inflammation. J. Nutr. Sci..

[36] Wilken R, Veena MS, Wang MB, Srivatsan ES (2011). Curcumin: a review of anti-cancer properties and therapeutic activity in head and neck squamous cell carcinoma. Mol. Cancer.

[37] Kunnumakkara AB, Bordoloi D, Harsha C, Banik K (2017). Curcumin mediates anticancer effect by modulating multiple cell signaling pathways. Clin Sci. (Lond).

[38] Sandur SK (2007). Curcumin, demethoxycurcumin, bisdemethoxycurcumin, tetrahydrocurcumin and turmerones differentially regulate anti-inflammatory and anti-proliferative responses through a ROS-independent mechanism. Carcinogenesis.

[39] Barrager E, Schauss AG (2003). Methylsulfonylmethane as a treatment for seasonal allergic rhinitis: additional data on pollen counts and symptom questionnaire. J. Altern. Complement. Med..

[40] Kim LS (2006). Efficacy of methylsulfonylmethane (MSM) in osteoarthritis pain of the knee: a pilot clinical trial. Osteoarthr Cartilage.

[41] Guimaraes-Stabili MR (2019). Systemic administration of curcumin or piperine enhances the periodontal repair: a preliminary study in rats. Clin Oral Investig.

[42] Srivastava NS, Srivastava RAK (2019). Curcumin and quercetin synergistically inhibit cancer cell proliferation in multiple cancer cells and modulate Wnt/beta-catenin signaling and apoptotic pathways in A375 cells. Phytomedicine.

[43] Chen L, Liu T, Wang Q, Liu J (2017). Anti-inflammatory effect of combined tetramethylpyrazine, resveratrol and curcumin *in vivo*. BMC Complement Altern Med.

[44] Choi H-J (2018). Development and validation of a HPLC-UV method for extraction optimization and biological evaluation of hot-water and ethanolic extracts of *Dendropanax morbifera* leaves. Molecules.

