# Supplementary material for: Anti-inflammatory Effect of Curcuma longa and Allium hookeri Co-treatment via NF-κB and COX-2 Pathways
Source: Sci Rep. 2020 Mar 31;10:5718. doi: 10.1038/s41598-020-62749-7 (PMC7109078; doi:10.1038/s41598-020-62749-7)

**Anti-inflammatory Effect of *Curcuma longa* and** ***Allium hookeri* Co-treatment via NF-κB and COX-2 Pathways**

Running title: Anti-inflammatory effect of *C. longa* and *A. hookeri* co-treatment

Soon-Young Lee^1,+^, Seung-Sik Cho^2,+^, YongChun Li^3^, Chun-Sik Bae^4^, Kyung Mok Park^1,*^, Dae-Hun Park^1,*^

^1^ Department of Korean Medicine, Dongshin University, Naju, 58245, Korea

^2^ Department of Pharmacy, College of Pharmacy, Mokpo National University, Muan Jeonnam 58579 Korea

^3^ School of Pharmaceutical Science, Zhengzhou University, Zhengzhou, Henan, 450001 P.R. China

^4^ College of Veterinary Medicine, Chonnam National University, Gwangju 61186 Korea

^*^corresponding: parkkim@dsu.ac.kr; [dhj1221@hanmail.net](mailto:dhj1221@hanmail.net)

^+^These authors contributed equally to this work


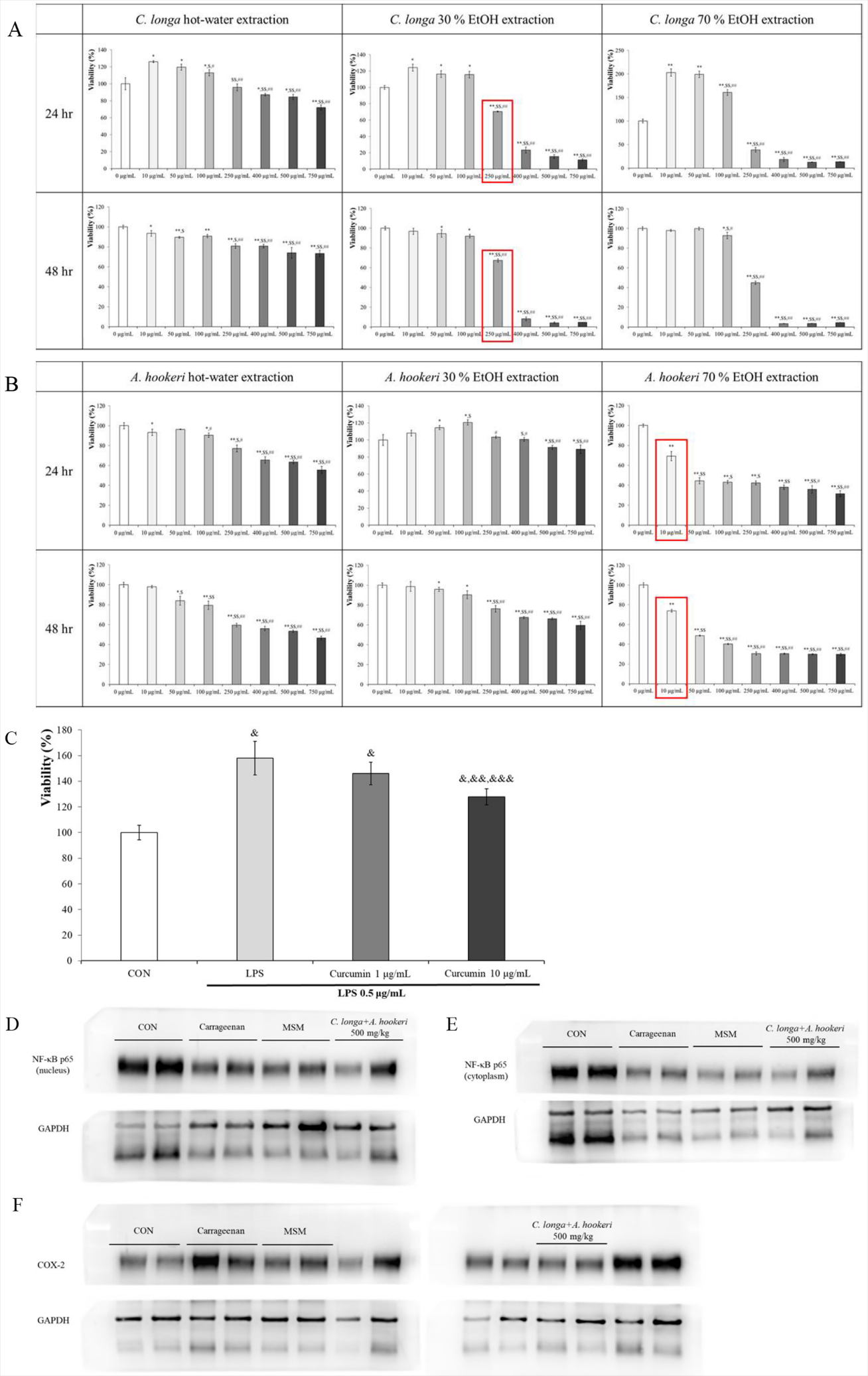

Supplement: Supplementary file 1 — Supplementary Information. [file 41598_2020_62749_MOESM1_ESM.docx]
