# Supplementary material for: Anti-inflammatory Effect of Curcuma longa and Allium hookeri Co-treatment via NF-κB and COX-2 Pathways
Source: Sci Rep. 2020 Mar 31;10:5718. doi: 10.1038/s41598-020-62749-7 (PMC7109078; doi:10.1038/s41598-020-62749-7)
Supplement: Supplementary file 2 — Supplementary Information2. [file 41598_2020_62749_MOESM2_ESM.docx]

**Supplementary File Legend**

According to incubation period and dosing, a percentage of live cells was treated with (A) *C. longa* and (B) *A. hookeri*. Red rectangles indicate the appropriate extract condition and dose. The extract condition and dose of *C. longa* or *A. hookeri* were 30% 250 μg/mL or 70% 10 μg/mL, respectively. The criteria for dose selection was that the dose should exhibit similar proliferation patterns for 24 h and 48 h incubation, and should limit about 20% cell proliferation that can be presumed as an unexcessive dose. (C) Cell viability depending on curcumin dose. (D) The levels of NF-κBp65 and GAPDH in nucleus. (E) The bands of NF-κBp65 and GAPDH in cytoplasm. (F) The bands of COX-2 and GPDH in whole proteins. All values are presented as mean ± standard deviation. 0 μg/mL vs. ^*^*p* <0.05; 0 μg/mL vs. ^**^*p* <0.001; 10 μg/mL vs. ^$^*p* <0.05; 10 μg/mL vs. ^$$^*p* <0.001; 50 μg/mL vs. ^#^*p* <0.05; 50 μg/mL vs. ^##^*p* <0.001; CON vs. ^&^*p* <0.001; LPS vs. ^&&^*p* <0.05; Curcumin 1 μg/mL vs. ^&&&^*p* <0.05.
